# TGF-β2 and collagen play pivotal roles in the spheroid formation and anti-aging of human dermal papilla cells

**DOI:** 10.18632/aging.203419

**Published:** 2021-08-17

**Authors:** Hyunju Kim, Nahyun Choi, Doo Yeong Kim, So Yoon Kim, Seung Yong Song, Jong-Hyuk Sung

**Affiliations:** 1 Epi Biotech Co., Ltd., Yeonsu-gu, Incheon 21984, South Korea; 2 College of Pharmacy, Institute of Pharmaceutical Sciences, Yonsei University, Yeonsu-gu, Incheon 21983, South Korea; 3 Department of Plastic and Reconstructive Surgery, Yonsei University College of Medicine, Seodaemun-gu, Seoul 03722, South Korea

**Keywords:** human dermal papilla cells, aggregation, collagen, TGF-beta2, anti-aging

## Abstract

Dermal papilla cells (DPCs) tend to aggregate both *in vitro* and *in vivo*, which increases the hair inductivity of DPCs. However, the underlying mechanism of spheroid formation is unknown. We investigated whether collagen expression in human DPCs (hDPCs) is involved in the spheroid formation and hair inductivity of hDPCs and further examined the underlying molecular mechanism of collagen upregulation. The expression of diverse collagens, such as COL13A1 and COL15A1, was upregulated in three dimensional (3D)-cultured or intact DPCs, compared to 2D-cultured hDPCs. This collagen expression was a downregulated in aged hair follicle*,* and aged DPCs were difficult to aggregate. Blocking of COL13A1 and COL15A1 by small interfering RNA reduced aggregation, while induced senescence of hDPCs *in vitro*. Further, transforming growth factor-β2 (TGF-β2) expression decreases with aging, and is involved in regulating the expression of COL13A1 and COL15A1. Addition of recombinant TGF-β2 delayed cellular senescence, and recovered spheroid formation in aged hDPCs by upregulating collagen levels. On the contrary, knock-out of TGF-β2 induced the aging of DPCs, and inhibited spheroid formation. These results suggested that COL13A1 and COL15A1 expression is downregulated with aging in DPCs, and upregulation of collagen by TGF-β2 induces the spheroid formation of DPCs. Therefore, TGF-β2 supplement in DPC culture medium could enhance the maintenance and hair inductivity of DPCs.

## INTRODUCTION

Hair follicle (HF) is composed of epidermal and dermal compartments, and their interaction plays an important role in HF morphogenesis and growth [1, 2]. The dermal papilla (DP), located at the base of the HF, is a unique tissue surrounded by epithelial matrix cells and is essential in controlling hair growth, formation, and cycling. Human DPCs (hDPCs) exhibit hair inductivity in the early passage and their hair inductivity is declined dramatically during aging. Therefore, there have been several efforts to induce the hair inductivity of hDPCs. For example, alkaline phosphatase (ALP) overexpression improves the hair inductive capability of cultured hDPCs [3], and hypoxic culture conditions induced the hair inductivity of hDPCs in the hair reconstitution assay [4]. Microenvironmental reprogramming by three-dimensional (3D) culture enables hDPCs to induce *de novo* human HF growth [5]. The tendency to aggregate is a significant characteristic of DPCs, and the ability to aggregate and hair inductivity are reduced after several passages of culture [1, 6]. The loss of the extracellular matrix (ECM) during culture could be responsible for the gradual loss of DPC inductive properties [7]; however, it has not been demonstrated.

The ECM is a complex and dynamic network of interacting fibrils and associated factors in intimate communication with cells [8, 9]. Although the specific role of the ECM in hair growth regulation is unknown, many studies in other biologic systems have shown that the ECM is involved in processes such as adhesion, cell proliferation, regulation of gene expression, and regulation of growth factor activity. In natural environments *in vivo*, DP is found at the HF base surrounded by a proteoglycan-rich ECM [10]. Therefore, it is reasonable to assume that DP interacts with ECM to induce the spheroid formation and regulates hair growth. Collagen is one of the major ECM components and one of the most abundant proteins in humans. Collagen functions are linked to many biological mechanisms involved in homeostasis maintenance and tissue development [11]. However, the involvement of collagen in the hair inductivity of hDPCs has not been determined.

In most cells, the fundamental functions of transforming growth factor-β (TGF-β) isoforms are growth control and ECM deposition [12]. Especially during fetal development, TGF-βs are found in a broad range of organs, such as epithelium, myocardium, cartilage, and bone of extremities, and the nervous system, suggesting its critical functions in organogenesis [13, 14]. In HF physiology, TGF-βs have been shown to exert unique multidirectional effects, such as inductive and suppressive effects on hair growth. For example, TGF-β1 blocks anagen and induces catagen [15], thereby inhibiting hair growth [16]. TGF-β2 induces premature HF regression in adult hair cycling [17, 18]. In contrast, TGF-β1 and TGF-β2 stimulate the proliferation of outer root sheath keratinocytes [19, 20]. It is of interest that TGF-β2 is required for hair folliculogenesis [21, 22]. Studies involving mice deficient in different TGF-β isoforms have shown that TGF-β2 is required for murine HF development, whereas TGF-β1 and TGF-β3 do not contribute significantly to this process [21]. TGF-β2 is primarily produced in the DP region and stimulates the proliferation of HF stem cells (HFSCs) by counteracting bone morphogenetic protein-mediated quiescence in the niche [23].

In this study, we investigated whether collagen expression changes in hDPCs are involved in the senescence and hair inductivity of hDPCs and further examined the underlying molecular mechanism of collagen regulation. This study reported that TGF-β2 inhibited the cellular senescence and induced spheroid formation of hDPCs by upregulating COL13A1 and COL15A1 expression.

## RESULTS

### Global gene expression profiles and functional analyses revealed the upregulation of collagen gene in hDPC aggregation culture

**Figure 1:**
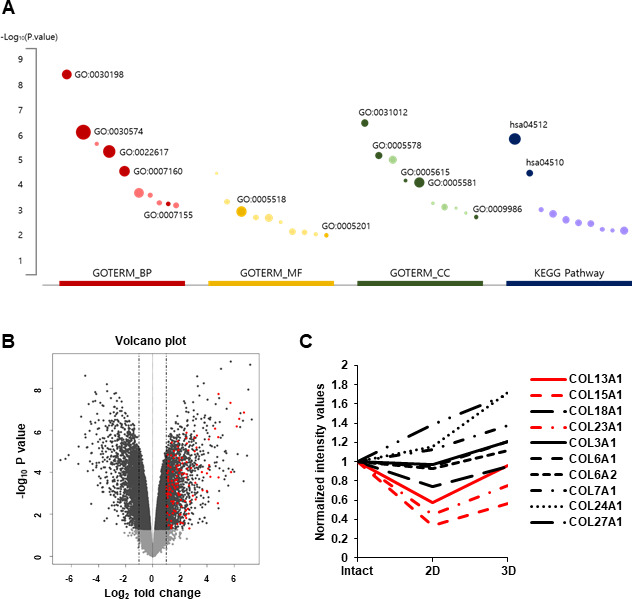
**RNA-seq analysis of 3D versus 2D hDPC cultures from previous report [5].** (**A**) Top 10 significantly enriched terms (*p* < 0.01) in GO and KEGG pathway for DEGs. The size of the dot indicates the enrichment fold of each term and pathway. Dark-colored dots are related to collagen or ECM. (**B**) Volcano plot of DEGs (|log_2_FoldChange| > 2 and adjusted *p* < 0.01). Red dots represent significantly upregulated ECM-related genes from GO and KEGG pathway analyses with DEGs. (**C**) Collagen gene expression. Each line represents a transcript differentially regulated among intact papillae (Intact), cells at P3 (2D), and spheroids (3D).

hDPCs gradually lose their proliferative capability and hair inductivity after passage *in vitro* [5, 24]. However, three-dimensional (3D) cultured DPCs showed higher hair inductivity than two-dimensional (2D) cultured DPCs and maintained hair inductivity at the old passage. The global gene expression profiles in hDPC aggregates were analyzed to investigate the superior effect of 3D cultured DPCs [5]. Genes whose expression levels were more than 1.5-fold (log_2_ fold change) higher in hDPC aggregates than those in hDPC monolayer cultures were extracted, and Gene Ontology (GO) enrichment analyses were performed on these genes. Data revealed that extracted genes were significantly enriched in ECM-related genes (Figure 1A). The functional annotation of differentially expressed genes (DEGs) related to fold change and *p* is presented in Supplementary Table 1. Moreover, a volcano plot was used to show the abundance of ECM-related genes in DEGs (Figure 1B). Among sets of gene groups under ECM-related genes, collagen-related genes were significantly upregulated in 3D cultured hDPCs compared to 2D cultured hDPCs. The top 10 upregulated collagen genes in 3D cultured hDPCs compared to 2D cultured hDPCs are displayed in Figure 1C. In particular, COL13A1, COL15A1, COL18A1, and COL23A1 transcripts were downregulated in 2D culture compared to intact papillae and 3D spheroids (Figure 1C). As COL18A1 expression is not downregulated at subsequent culture passages (P0, P1, P3, and P5) contrary to COL13A1, COL15A1, and COL23A1 (Supplementary Figure 1A–1D), whether COL13A1, COL15A1, and COL23A1 can affect aggregation and cellular senescence of hDPCs was further studied.

### COL13A1 and COL15A1 contributed to the aggregation and cellular senescence of hDPCs

**Figure 2:**
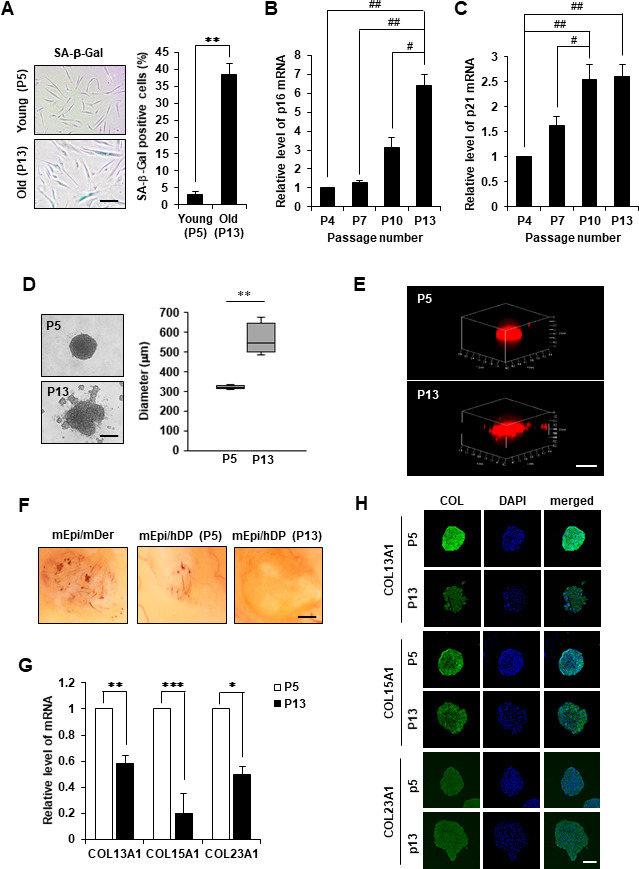
**Cell aggregation, HF induction ability, and collagen expression of replicative senescent hDPCs.** (**A**) SA-β-gal staining in young (P5) and old (P13) passage cells. Scale bar, 200 μm. Quantification for n > 200 cells per group. p16 (**B**) and p21 (**C**) mRNA expression by qRT-PCR. (**D**) Representative image of spheroids from young (P5) or old (p13) passage cells is shown on (top), and the size in diameter of spheroids (μm) is quantified (right). Scale bar, 200 μm. (**E**) 3D reconstruction of spheroids. Scale bar, 500 μm. (**F**) Patch assay. Senescent hDPCs (P13) failed to induce new HFs (n = 3). Scale bar, 500 μm. (**G**) COL13A1, COL15A1, and COL23A1 mRNA expression by qRT-PCR. (**H**) Immunofluorescence staining of hDPC spheroids with COL13A1, COL15A1, COL23A1, and DAPI for nuclei. Scale bar, 200 μm. All quantitative data are shown as the mean ± standard deviation (SD) of three independent experiments. *p < 0.05; **p < 0.01; ***p < 0.005; ^#^*p* < 0.05; ^##^*p* < 0.01. Asterisk indicates Student's *t*-test. Sharp indicates one-way ANOVA.

First, the cellular phenotype of aged hDPCs was analyzed by the morphological change and the expression of classical senescence-associated markers, such as β-galactosidase (β-gal), p16 and p21 from the early passage (P5) to the senescent passage (P13). Senescent hDPCs exhibited morphological changes, including enlarged size and flattened shape, and enhanced β-gal activity (Figure 2A). Senescent hDPCs also showed increased p16 and p21 mRNA expression compared to early-passage hDPCs (Figures 2B, 2C).

Next, whether cellular senescence of hDPCs could affect their aggregation potential was investigated using a 3D culture system. hDPCs (10,000 cells/well) became spheroids with diameter ranging from 300 to 600 μm after 24 h incubation (Figure 2D). Young hDPCs (P5) generated round and compact spheroids with tight cell adhesion, whereas senescent hDPCs (P13) formed spheroids with an unstructured outline and weak cell-cell adhesion (Figure 2D). 3D reconstructed confocal scanning microscopy images further illustrated distinct structural differences among spherical colonies produced between young and senescent 3D spheroidal hDPCs (Figure 2E). Moreover, young hDPCs induced HF regeneration, whereas aged hDPCs could not regenerate HF in the hair reconstitution assay (Figure 2F). These results indicated that senescent hDPCs exhibit reduced aggregative activity, which might contribute to low hair inductivity.

**Figure 3:**
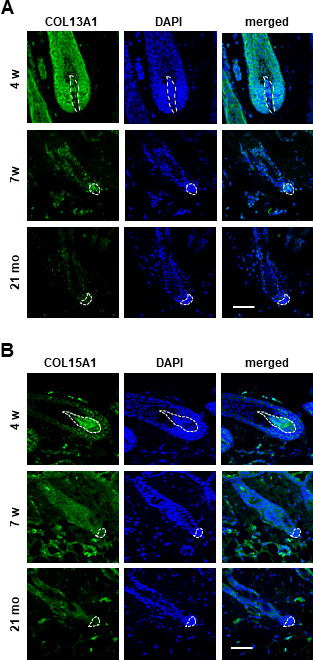
**The expression of COL13A1 and COL15A1 were reduced in DPC of mice HF.** Skin biopsies of normal C57BL/6 mice were collected at the indicated age and processed for paraffin sections. COL13A1 (**A**) and COL15A1 (**B**) expression was visualized by immunofluorescence staining and counterstained with DAPI for nuclei. The DP was circled by white dashed lines in each HF. Scale bar, 50 μm.

Further, significant downregulation of COL13A1, COL15A1, and COL23A1 mRNA levels (Figure 2G) compared to COL3A1, COL6A1, COL6A2, COL7A1, COL18A1, COL24A1, and COL27A1 (Supplementary Figure 1E) was confirmed in senescent hDPC spheroids. However, immunostaining results showed that the levels of COL13A1 and COL15A1 protein expression were decreased in 3D spheroids of senescent hDPCs but not COL23A1 (Figure 2H and Supplementary Figure 1F). We confirmed that COL13A1 and COL15A1 expression was reduced in senescent hDPCs through western blot (Supplementary Figure 1G). Because the expression levels of COL13A1 and COL15A1 were decreased in senescent hDPCs, COL13A1 and COL15A1 expression in HF of young (4 and 7 weeks) and old (21 months) mice was investigated. Aged HFs were positive for senescence-associated β-gal (SA-β-gal) at the secondary germ and DP of the telogen HF (Supplementary Figure 2), indicating HF senescence. The expression levels of COL13A1 and COL15A1 were significantly decreased in DP of aged mice (Figure 3A, 3B). These results indicated that senescence of DPCs is associated with low expression levels of COL13A1 and COL15A1.

### The knockdown of COL13A1 and COL15A1 induced cellular senescence and reduced aggregation in hDPCs

**Figure 4:**
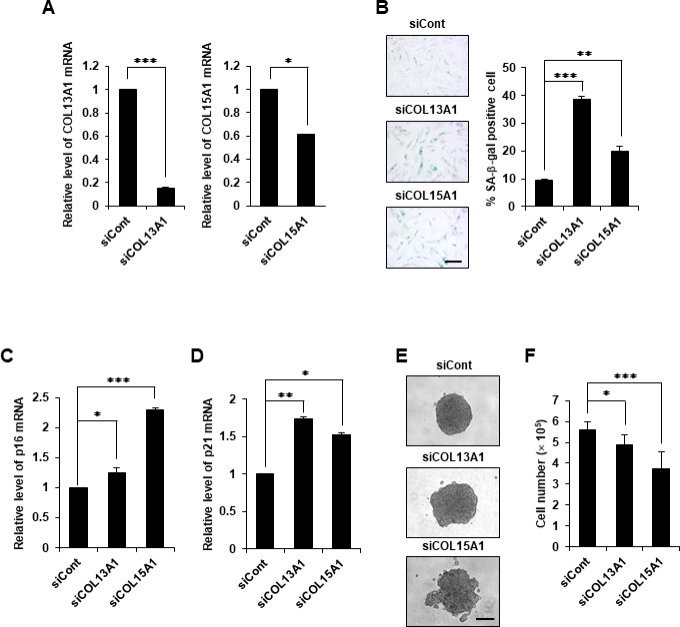
**COL13A1 and COL15A1 knockdown induced senescence in hDPCs.** (**A**) hDPCs (P5) were transfected with COL13A1 (siCOL13A1), COL15A1 (siCOL15A1), or control (siCont) siRNAs for 48 h, after which the amounts of COL13A1 (left) and COL15A1 (right) mRNA were determined by qRT-PCR. (**B**) Quantification and representative images of SA-β-gal-positive cells in control, COL13A1, and COL15A1 siRNA-treated hDPCs (P5). Scale bar, 200 μm. p16 (**C**) and p21 (**D**) mRNA expression by qRT-PCR after siRNA transfection for 48 h. (**E**) hDPCs (P4) were transfected with COL13A1- or COL15A1-specific siRNA for 24 h and then grown in 3D for another 24 h. Representative images for hDPC spheroids transfected with control, COL13A1, or COL15A1 siRNA. Scale bar, 200 μm. (**F**) Cell numbers after COL13A1 and COL15A1 siRNA transfection for 48 h. All quantitative data are shown as the mean ± SD of three independent experiments. *p < 0.05; **p < 0.01; ***p < 0.005. Asterisk indicates Student's *t*-test.

To reveal whether COL13A1 and COL15A1 play pivotal roles in the aggregative behavior and cellular senescence of DPCs, the levels of COL13A1 and COL15A1 were reduced using small interfering RNA (siRNA). The reduced expression levels of COL13A1 and COL15A1 mRNA were confirmed by quantitative real-time reverse transcription-polymerase chain reaction (qRT-PCR; Figure 4A). Indeed, the knockdown of COL13A1 and COL15A1 induced cellular senescence of hDPCs as evidenced by the increased SA-β-gal activity (Figure 4B) and the upregulated p16 and p21 mRNA expression (Figure 4C, 4D). hDPCs transfected with control siRNA formed compact 3D spheroids, whereas hDPCs transfected with COL13A1 and COL15A1 siRNAs did not form compact 3D spheroids (Figure 4E). In particular, hDPCs transfected with COL13A1 and COL15A1 showed reduced proliferation activity (Figure 4F). Moreover, hDPCs transfected with COL13A1 and COL15A1 siRNAs showed significantly reduced HF regeneration (Supplementary Figure 3). These results suggested that COL13A1 and COL15A1 play key roles in maintaining hair inductivity of DPCs and preventing senescence.

### TGF-β2 induced aggregation and delayed senescence by regulating COL13A1 and COL15A1 expression

**Figure 5:**
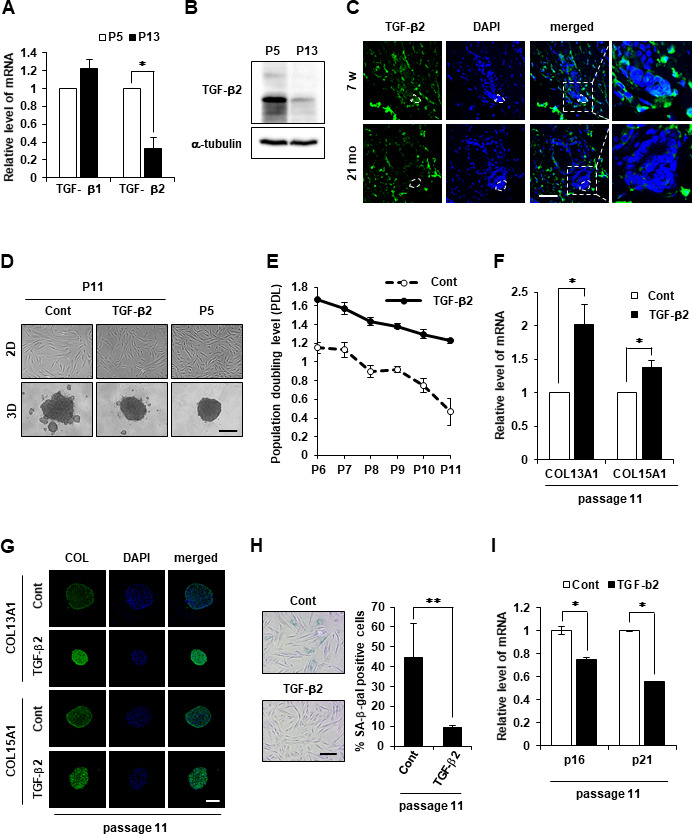
**TGF-β2 supplement maintains cell aggregative behavior and prevents senescence in DPCs.** (**A**) TGF-β1 and TGF-β2 mRNA expression was determined by qRT-PCR in P5 and P13 DPC spheroids. (**B**) TGF-β2 expression was determined by western blotting in P5 and P13 DPC spheroids. (**C**) Skin biopsies of normal C57BL/6 mice were collected at the indicated age and processed for paraffin sections. TGF-β2 expression was visualized by immunofluorescence staining and counterstained with DAPI for nuclei. The DP was circled by white dashed lines in each HF. Scale bar, 50 μm. (**D**) Representative image of 2D and 3D spheroids of hDPCs cultured in the absence or presence of TGF-β2. Cells were sustained with TGF-β2 (50 ng/ml) in the culture medium from P5 to P11, and the total cell lysates from P11 were used. Scale bar, 200 μm. (**E**) Cell duplication level between consecutive passages of DPCs in the presence or absence of TGF-β2 (50 ng/ml) was investigated by cell counting. To calculate the duplication level of each passage, the harvested cell number was normalized to the seeding cell number at a 2-day interval. Experiments were carried out in triplicates. COL13A1 and COL15A1 mRNA expression by qRT-PCR (**F**) and immunofluorescence image (**G**) in 3D spheroids at P11 of hDPCs cultured in the absence or presence of TGF-β2. Scale bar, 200 μm. (**H**) Quantification and representative images of SA-β-gal-positive cells in P11 of hDPCs cultured in the absence or presence of TGF-β2. (**I**) p16 and p21 mRNA expression by qRT-PCR in P11 of hDPCs cultured in the absence or presence of TGF-β2. All quantitative data are shown as the mean ± SD of three independent experiments. *p < 0.05; **p < 0.01. Asterisk indicates Student's *t*-test.

It was hypothesized that TGF-βs may contribute to COL13A1 and COL15A1 expression. The downregulation of TGF-β2 mRNA was also detected in senescent hDPCs, whereas no significant change in TGF-β1 mRNA expression was found (Figure 5A). The level of TGF-β2 protein was also downregulated in senescent hDPCs (Figure 5B). A significantly lower level of TGF-β2 was detected in the DP region of aged HF (21 months) compared to young mice HFs (7 weeks; Figure 5C). Next, to investigate whether TGF-β2 supplement could induce compact 3D spheroids of hDPCs even in old passage, hDPCs have been cultured with or without TGF-β2 from P5 to P11. TGF-β2 treatment in hDPCs not only increased cell proliferation evidenced by population doubling but also restored the aggregative ability of hDPCs to the level of young cells (Figure 5D, 5E). The mRNA and protein expression levels in aged hDPCs were examined to further verify whether collagen expression was regulated by TGF-β2. Both mRNA and protein levels of COL13A1 and COL15A1 were upregulated by TGF-β2 treatment in aged DPCs (Figure 5F, 5G, and Supplementary Figure 4A, 4B). It was also investigated whether TGF-β2 treatment could recover aged DPCs to young phenotype. Indeed, TGF-β2 treatment reduced the senescent phenotype of hDPCs as evidenced by β-gal activity and p16 and p21 expression (Figure 5H, 5I). These results indicated that TGF-β2 signaling plays a role in aggregative ability and anti-aging of hDPCs by regulating COL13A1 and COL15A1 expression. Next, we investigated whether delaying the aging of hDPC by treatment with TGF-β2 can promote hair growth. We found that injection of naïve (untreated) hDPCs (P12) increased telogen-to-anagen induction in mice only slightly following subcutaneous injection, while TGF-β2-treated hDPCs (P12) induced robust hair growth (Supplementary Figure 4C, 4D). Hair growth by TGF-β2-treated hDPC (P12) was similar to that of naïve young hDPC (P5). This result suggested that delayed aging of hDPC by TGF-β2 treatment restored the hair growth effect of hDPC to a youthful state.

### TGF-β2 knockout (KO) reduced aggregation and induced DPC senescence

**Figure 6:**
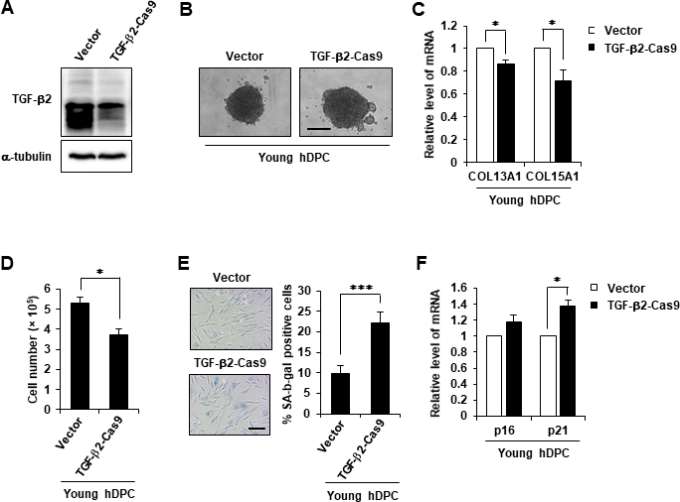
**TGF-β2 KO induced cellular senescence.** (**A**) Western blot analysis of TGF-β2 in TGF-β2 knockout hDPCs (P5). (**B**) Representative image of hDPC spheroids transfected with control or TGF-β2 CRISPR/Cas9 KO plasmid for 48 h. (**C**) COL13A1 and COL15A1 mRNA expression in 3D spheroids of CRISPR/Cas9 KO plasmid-transfected hDPC. (**D**) Cell numbers after control or TGF-β2 CRISPR/Cas9 KO plasmid transfection for 48 h. (**E**) Quantification and representative images of SA-β-gal-positive cells in control and TGF-β2 CRISPR/Cas9 KO plasmid-treated hDPCs. Scale bar, 200 μm. (**F**) p16 and p21 mRNA expression by qRT-PCR after CRISPR/Cas9 KO plasmid transfection for 48 h. All quantitative data are shown as the mean ± SD of three independent experiments. *p < 0.05; ***p < 0.005. Asterisk indicates Student's *t*-test.

Next, it was confirmed whether TGF-β2 inhibition accelerated the aging-related phenotype. CRISPR/Cas9-mediated TGF-β2 KO was performed in early-passage hDPCs, and CRISPR/Cas9-mediated TGF-β2 KO was confirmed (Figure 6A). The 3D spheroid formation assay revealed that TGF-β2 KO reduced the aggregative behavior and cell growth of hDPCs and downregulated COL13A1 and COL15A1 expression (Figure 6B–6D). TGF-β2 KO also induced the senescent phenotype as evidenced by a substantial increase of β-gal activity and p21 mRNA upregulation (Figure 6E, 6F).

## DISCUSSION

DPCs tend to aggregate, which is related to the hair inductivity of DPCs. Therefore, DPCs need to be cultured in 3D culture to create mimetic physiological cell state [6]. This study was investigated whether collagen expression changes in hDPCs are involved in the spheroid formation and hair inductivity of hDPCs and further examined the underlying molecular mechanism of collagen regulation. The expression of diverse collagen, such as COL13A1 and COL15A1, was upregulated in 3D compared to 2D cultured hDPCs. Their expression was also downregulated during aging. In addition, COL13A1 and COL15A1 were expressed in the DP region of young mice (4 weeks and 7 weeks) HF, and this expression in DPC was decreased in old mice (21 months). Blocking of COL13A1 and COL15A1 by siRNA reduced aggregative behavior and induced senescence of hDPCs *in vitro*. Further, TGF-β2 played a key role in COL13A1 and COL15A1 regulation. TGF-β2 upregulated collagen levels, delayed cellular senescence, and induced aggregation capacity in hDPCs.

Higgins et al. reported that intact DP transcriptional signature could be partially restored by 3D spheroid cultures of hDPCs [5]. They also reported the expression changes in several transcripts [clusterin (CLU), endothelin 3 (EDN3), low-density lipoprotein receptor-related protein 4 (LRP4), Wnt inhibitory factor 1 (WIF1), adenomatosis polyposis coli downregulated 1 (APCDD1), lymphoid enhancer-binding factor 1 (LEF1), chemokine (C-X-C motif) receptor 4 (CXCR4), matrix gla protein (MGP), and gremlin 1 (GREM1)], including several transcripts known to play an important role in HF generation. They mainly focused on signature transcripts that changed when DPC is cultured in 3D condition compared to 2D [5]. Among those differentially expressed genes, we were particularly interested in ECM proteins and reanalyzed their microarray data focusing on ECM proteins. Diverse collagen (>10 types) was highly expressed in 3D spheroid cultures of hDPCs compared to 2D cultures. Among them, we found that COL13A1 and COL15A1 play key roles in the spheroid formation and hair inductivity of DPCs.

One cellular phenomenon associated with the deficiency of ECM components is cellular senescence. In addition, a defect of collagen levels might stimulate the beginning of cellular senescence. Previous study revealed that COL17A1 is critical for the self-renewal of HFSCs through maintaining their quiescence and immaturity [25]. Reduced COL17A1 levels in the niche led to a loss of stemness of HFSCs resulting to the depletion of the stem cell pool [25]. The aging process can be accelerated in COL17A1-deficient mice, and forced expression of COL17A1 rescued premature differentiation of stem cell and TGF-β signaling suggesting that HFSC aging is mediated by the niche ECM, such as collagen. Our data revealed that COL13A1 or COL15A1 expression is reduced in aged DPC spheres and in DPC of aged mice HF (Figures 2, 3). Also, collagen knockdown induced cellular senescence and reduced DPC aggregation, which in turn reduced hair inductivity (Figure 4 and Supplementary Figure 3). These results suggested that the maintenance of collagen expression in hDPCs is critical for anti-aging and hair inductivity of DPC. In addition to COL17A1 in the epidermal region of HF, expression of COL13A1 or COL15A1 in DPC is very important in self-aggregation and preventing cellular senescence of HF.

Next, we assumed that TGF families might play a key role in collagen expression. Therefore, the involvement of TGF-β1 or TGF-β2 in senescence of DPCs and collagen expression was investigated. TGF-β1 expression was not reduced in aged hDPCs (Figure 5A). However, TGF-β2 expression was significantly decreased in aged hDPCs (Figure 5A, 5B) and in DPC of aged mice HF (Figure 5C). Moreover, TGF-β2 treatment increased the expression of COL13A1 and COL15A1 in aged hDPCs (Figure 5F, 5G). TGF-β2 treatment also induced the aggregation of DPCs in 3D culture and inhibited the aging of DPCs (Figure 5D–5I), whereas TGF-β2 KO exhibited negative effects on DPC maintenance (Figure 6). These results are consistent with previous studies in which TGF-β2 is synthesized in the DP and required for DPC maintenance [21, 22]. For example, Inoue et al. compared the signature genes of hDPCs and human dermal fibroblasts, and found that TGF-β2 is highly expressed in cultured hDPCs [26]. Pharmacological inhibition and neutralizing antibody treatment of the TGF-β2 signaling pathway inhibited HF generation in animal models [26]. Vitamin D3 analog was found to promote TGF-β2 expression and ALP activity of hDPCs to enhance hair inductivity [27]. Similarly, we also found that TGF-β2-treated hDPCs had a hair promoting effect compared to the untreated cells (Supplementary Figure 4C, 4D) suggesting that delayed aging of hDPC by recombinant TGF-β2 treatment restored the hair growth effect of hDPCs. Although Inoue et al. did not measure the change in collagen expression by TGF-β2, our study suggested that TGF-β2 upregulation in hDPCs might enhance the hair inductivity of hDPCs through induction of collagen expression [26].

TGF-β2 signaling have been reported for involved in various types of cancer [28, 29]. So, we examined the expression of oncogenes such as Snail, Slug, HRAS, KRAS, and Raf-1 in hDPCs after TGF-β2 treatment. However, TGF-β2 itself did not show any stimulating effect on the expression of oncogene in hDPCs (Supplementary Figure 5).

**Figure 7:**
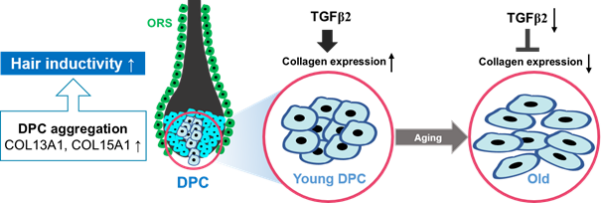
**A proposed scheme for the involvement of TGF-β2 and collagen in the aggregative behavior and anti-aging of hDPCs.** COL13A1 and COL15A1 upregulation by TGF-β2 increases the spheroidal formation of DPCs, thereby reducing cellular senescence and inducing the hair inductivity of DPCs.

As summarized in Figure 7, COL13A1 and COL15A1 upregulation by TGF-β2 increases the spheroidal formation of DPCs, thereby reducing cellular senescence and inducing the hair inductivity of DPCs.

Therefore, TGF-β2 supplement in DPC culture medium or collagen coating in plastic dishes could enhance the maintenance and hair inductivity of DPCs.

## MATERIALS AND METHODS

### DEGs from 2D cultured DPCs and 3D cultured spheroid DPCs

To compare the transcriptional profiles of 2D cultured DPCs and 3D cultured spheroid DPCs, microarray data (GSE44765) were used from the National Center for Biotechnology Information Gene Expression Omnibus database. For bioinformatic analysis, data were processed with R studio using Limma package. Uncharacterized and undefinable genes were deleted from the list. Genes that were upregulated > ***log_2_ (fold change) > 1.5***, adjusted *p* < 0.01 were considered statistically significant for upregulated DEGs.

### GO biological process (BP), molecular function (MF), cellular component (CC), kyoto encyclopedia of genes and genomes (KEGG) pathway enrichment analyses, and volcano plot

GO BP, MF, and CC terms that annotate a list of enriched genes and KEGG pathway statistical enrichment of DEGs was performed using the David Bioinformatics Resources 6.8 NIAID/NIH (https://david.ncifcrf.gov/; *p* < 0.01). A total of 635 upregulated DEG symbols have been put into this analysis tool. A plot that presented the top 10 terms with -logP values(y-axis) and enrichment fold (area of the dot) was made using the R package and then retouched using Adobe Illustrator. A volcano plot was plotted using R Studio, Deseq package.

### Cell culture

hDPCs at P5 to P13 were obtained (c-12071; PromoCell, Heidelberg, Germany) and cultured in Follicle DPC Growth Medium (PromoCell) with 0.1% Gibco^™^ antibiotic-antimycotic (Thermo Fisher Scientific, Waltham, MA, USA). Cells were maintained in a humidified incubator at 37° C under 5% CO_2_.

### SA-β-gal assay

hDPCs (1 x 10^5^) were seeded in 30 mm dishes with a growth medium. SA-β-gal activity was determined using a histological staining kit according to the manufacturer’s instructions (Cell Signaling).

### qRT-PCR assay

Total cellular RNA was extracted using Invitrogen TRIzol reagent (Thermo Fisher Scientific), followed by reverse transcription using a cDNA synthesis kit (Nanohelix, Daejeon, Korea). qRT-PCR was performed using the StepOne Real-Time PCR System (Applied Biosystems/Thermo Fisher Scientific). The primer sequences used are as follows (forward and reverse, respectively): 5′-TGGAGAACAGGGACCAGATGGC-3′ and 5′-GATCTCCTGGAGAGCCTCATTG-3′ for COL13A1, 5′-GGTGACACTGGTTTACCTGGCT-3′ and 5′-GCCTTTCCAGAGGAATGTCCTC-3′ for COL15A1, 5′-CTCGTGCTGATGCTACTGAGGA-3′ and 5′-GGTCGGCGCAGTTGGGCTCC-3′ for p16, and 5′-AGGTGGACCTGGAGACTCTCAG-3′ and 5′-TCCTCTTGGAGAAGATCAGCCG-3′ for p21.

### Generation of DPC spheroids

For spheroid generation, 100 μl/well of cell suspensions at 1 x 10^5^ cells/ml were dispensed into ultra-low attachment 96-well round-bottomed plates (Corning B.V. Life Sciences, Amsterdam, The Netherlands) using a multichannel pipette. Plates were incubated for 24 h at 37° C under 5% CO_2_. Images were captured using a microscope (Nikon) equipped with a CCD camera and imported into HKBasic software.

### 3D image visualization

hDPCs were tagged with PKH26 Red Fluorescent Cell Linker Kit (Sigma-Aldrich, St. Louis, MO, USA) at room temperature for 5 min in the dark and blocked with fetal bovine serum according to the manufacturer’s instructions. Cells were seeded into ultra-low attachment 96-well round-bottomed plates (Corning B.V. Life Sciences) at 1 x 10^4^ per well. A laser scanning confocal fluorescence microscope was used to obtain Z-stacked images of PKH26-stained cells. 3D images were constructed by ZEN 2011 software.

### Immunofluorescence staining

The paraffin sections were dewaxed three times using xylene for 15 min and hydrated in 100%, 90%, 80%, and 70% ethanol. Antigen retrieval was performed using a microwave oven with boiling antigen retrieval solution (pH 6.0; Dako, Carpinteria, CA, USA) for 2 min 20 s. The sections were stained with rabbit COL13A1 (1:100; MyBioSource, San Diego, CA, USA), rabbit COL15A1 (1:100; MyBioSource), and mouse TGF-β2 (1:100; Santa Cruz Biotechnology, Dallas, TX, USA) antibodies overnight at 4° C and then incubated with Alexa Fluor 488 goat anti-rabbit immunoglobulin G (IgG; 1:200; Invitrogen, Grand Island, NY, USA) or Alexa Fluor 488 goat anti-mouse IgG (1:200; Invitrogen) for 1 h at room temperature with 4′,6-diamidino-2-phenylindole (DAPI; Sigma-Aldrich). For cell staining, cells were fixed with 4% paraformaldehyde for 30 min at room temperature, washed with phosphate-buffered saline, and incubated with TGF-β2 antibodies overnight at 4° C. The samples were then incubated with Alexa Fluor 594 goat anti-mouse IgG (1:1000; Invitrogen) secondary antibodies for 1 h at room temperature with DAPI. Images of immunofluorescence staining were captured using a Zeiss LSM700 confocal microscope.

### Western blotting

For the preparation of whole-cell extracts, adherent cells were washed by PBS, removed by scraping, and lysed in RIPA lysis buffer (Biosesang, Seongnam, Gyeonggi, Korea). Total protein was separated by sodium dodecyl sulfate-polyacrylamide gel electrophoresis (SDS-PAGE) using 10% or 12% gels and transferred to PVDF membranes (Millipore, Bedford, MA, USA). Membranes were blocked with 5% fat-free dried milk in TBS-T (0.1% Tween 20 in Tris-buffered saline) for 1 h at room temperature and then incubated overnight with primary antibody at 4° C. The following day, the membranes were washed thrice with TBS-T and incubated with HRP-conjugated secondary antibody for 1 h at room temperature. The membrane was then reacted with enhanced chemiluminescence solution (Millipore) and photographed.

### Cell growth

Cells were transfection and incubated for 48 h. To measure cell growth, treated cells were trypsinized and counted using the trypan blue exclusion method.

### Skin reconstitution assay (patch assay)

To determine the hair inductivity of hDPCs, an established patch assay was performed [30]. Briefly, freshly isolated epidermal cells were obtained from newborn (C57BL/6 mice) epidermis. Next, hDPC spheroids (each with ~100 DPCs at P5 and P13) harvested from ultra-low attachment 96-well round-bottomed plates were combined with 1 x 10^6^ epidermal cells and subcutaneously implanted into the dorsal side of 6-week-old nude mice (BALB/cAJcl-nu). Dermal cells mixed with epidermal cells (1 x 10^6^) were used as the control. After 3 weeks of implantation, all animals were sacrificed. For macroscopic observation, dissected samples were first characterized and imaged under a stereoscope.

### Population doubling level

hDPCs were seeded in 60 mm dishes at 2 x 10^5^ and passaged every 2 days. For every passage, cells were trypsinized, stained with trypan blue (Sigma-Aldrich), and counted using a hemocytometer. Cell numbers at the start of each passage and after harvest were used to calculate the number of population doubling using the following formula: PDL = log_2_ (number of harvested cells)/(number of seeded cells), where PDL = population doubling level and log_2_ = natural logarithm of 2.

### Mouse telogen-to-anagen induction

The mice were maintained and anesthetized according to a protocol approved by the US Pharmacopeia and the Institutional Animal Care and Use Committee of Yonsei University (IACUC-A-202004-1052-02). The dorsal region of 7-week-old male C3H/HeN mice in the telogen stage of the hair cycle was shaved and depilated using an electric shaver, with special care taken to avoid damaging the bare skin. For TGF-β2-treated DPC injection experiment, 4 × 10^4^ of the control DPC or TGF-β2 (50 ng/mL)-treated DPC for 5~12 passage were injected into the dorsal skin of the shaved mice. After the mice was first shaved, the actual length of the shaved part was measured. Almost all mice were shaved with size of approximately 2 × 4.3 cm, and only the weight of new hair in the shaved area was measured after sacrifice. Any darkening of the skin (indicative of hair cycle induction) was carefully monitored using image capture. After approximately 14 days, the dorsal hair was shaved and weighed to estimate growth rate.

### CRISPR/Cas9 transfection

For TGF-β2 KO, hDPCs were seeded in 6-well plates. On the following day, 1 μg control or TGF-β2 CRISPR/Cas9 KO plasmid (Santa Cruz Biotechnology, Santa Cruz, CA, USA) were transfected using Lipofectamine 2000 (Invitrogen, Waltham, MA, USA). Cells were incubated for 72 h after transfection, and TGF-β2 silencing outcomes were evaluated using immunofluorescence staining.

### Data analysis and statistics

Data are presented as the mean ± standard deviation of three independent experiments. Student's *t*-test was used between two groups and one-way analysis of variance with Tukey's post hoc test were used for comparing multiple groups. *P* < 0.05 was considered to indicate a statistically significant difference. All statistical analyses were conducted using GraphPad Prism 5.01 (GraphPad Software, Inc., La Jolla, CA, USA).

## Supplementary Material

Supplementary Figures

Supplementary Table 1
