# Supplementary material for: TGF-β2 and collagen play pivotal roles in the spheroid formation and anti-aging of human dermal papilla cells
Source: Aging (Albany NY). 2021 Aug 17;13(16):19978–95. doi: 10.18632/aging.203419 (PMC8436940; doi:10.18632/aging.203419)
Supplement: Supplementary Figures [file aging-13-203419-s001.pdf]

SUPPLEMENTARY FIGURES

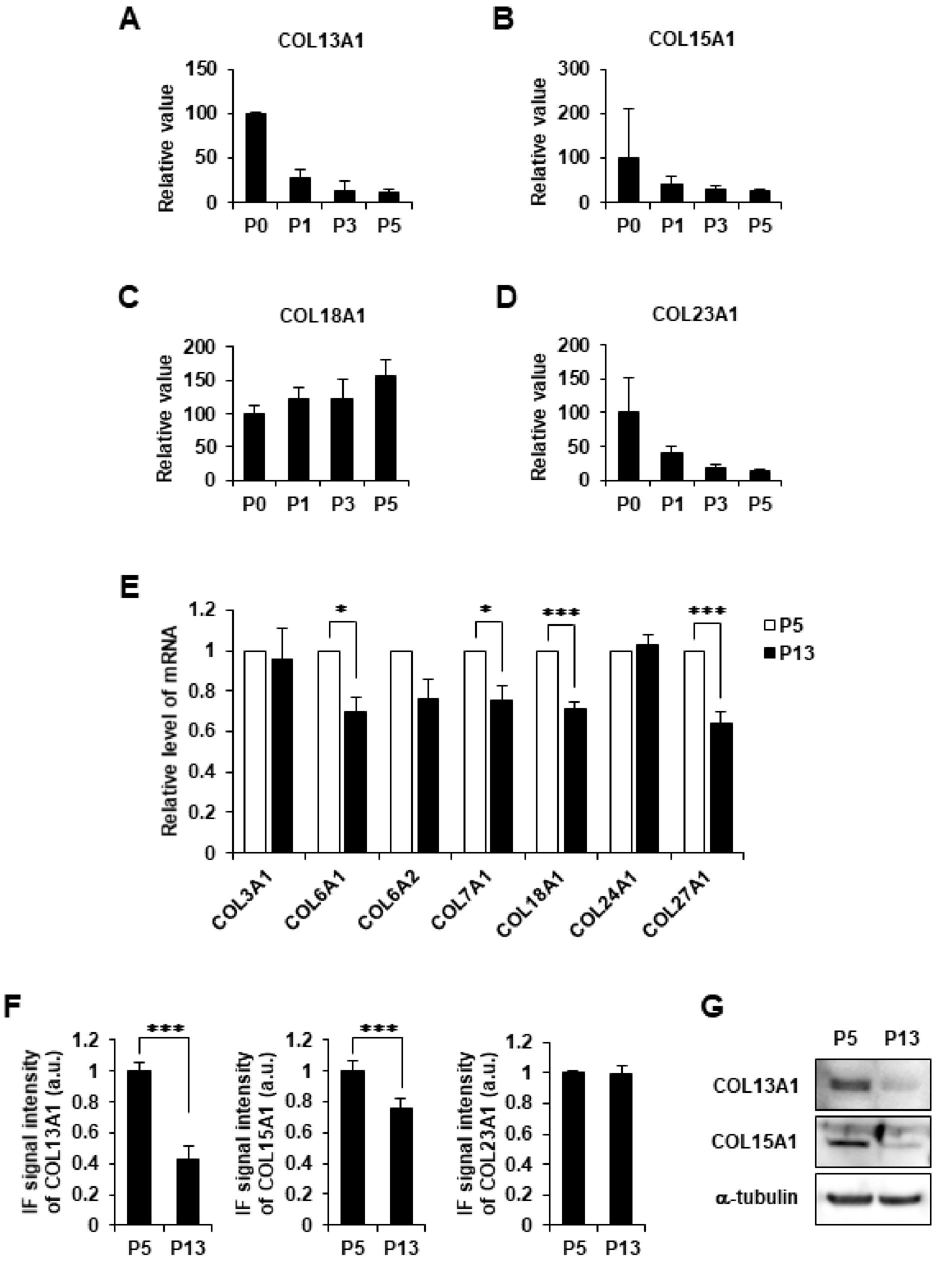

Supplementary Figure 1. The expression of COL13A1, COL15A1, COL18A1, and COL23A1. (**A**–**D**) Transcript levels of selected collagen genes at subsequent culture passage (P0, P1, P3, and P5). (**E**) qRT-PCR analysis of collagen mRNAs in 3D cultured hDPCs (P5 and P13). (**F**) Quantification of COL13A1, COL15A1, and COL23A1 signal intensity in 3D spheroid. (**G**) The expression of COL13A1 and COL15A1 was determined by Western blotting. Equal protein loading was verified by α-tubulin. *$p < 0.05$; ***$p < 0.005$. Asterisk indicates Student's *t*-test.

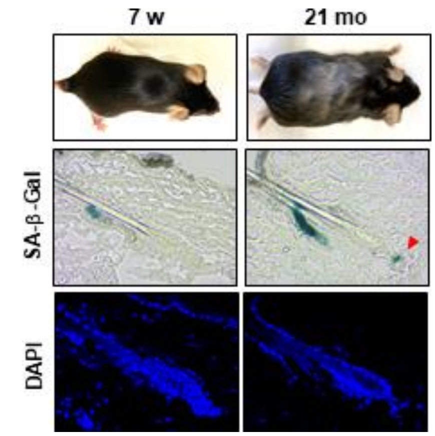

Supplementary Figure 2. Young and aged telogen HFs stained for SA-β-Gal. Red arrows indicate high SA-β-Gal expression in the secondary germ and DP.

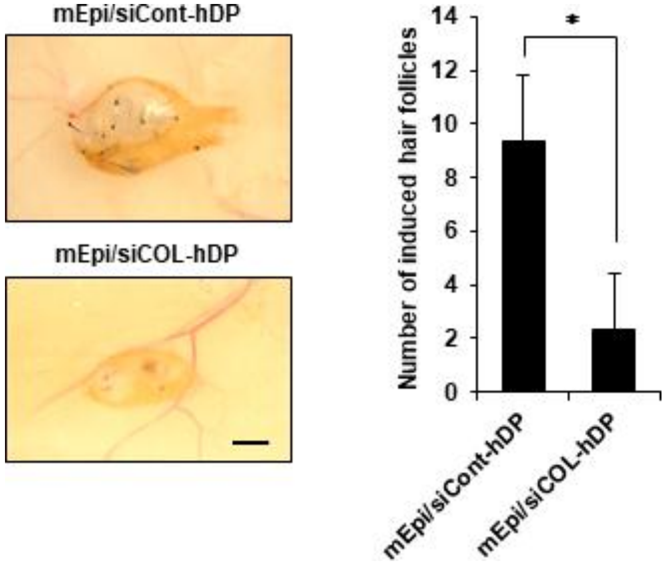

Supplementary Figure 3. Impairment of hair inductive activity of hDPCs by knockdown of COL13A1 and COL15A1. HFs were ordinarily induced when control siRNA-transfected hDPC spheres (10^6 cells; 100 DP spheres) were implanted together with mouse epidermal cells (10^6 cells). HF induction was severely impaired when COL13A1 and COL15A1 siRNA-transfected hDPC spheres (10^6 cells; 100 DP spheres) and mouse epidermal cells (10^6 cells) were cotransplanted. Scale bar, 500 µm. Total induced HFs were counted at each injection site, and data are means ± SD of triplicate per experiment from three independent experiments. *$p < 0.05$. Asterisk indicates Student's *t*-test.

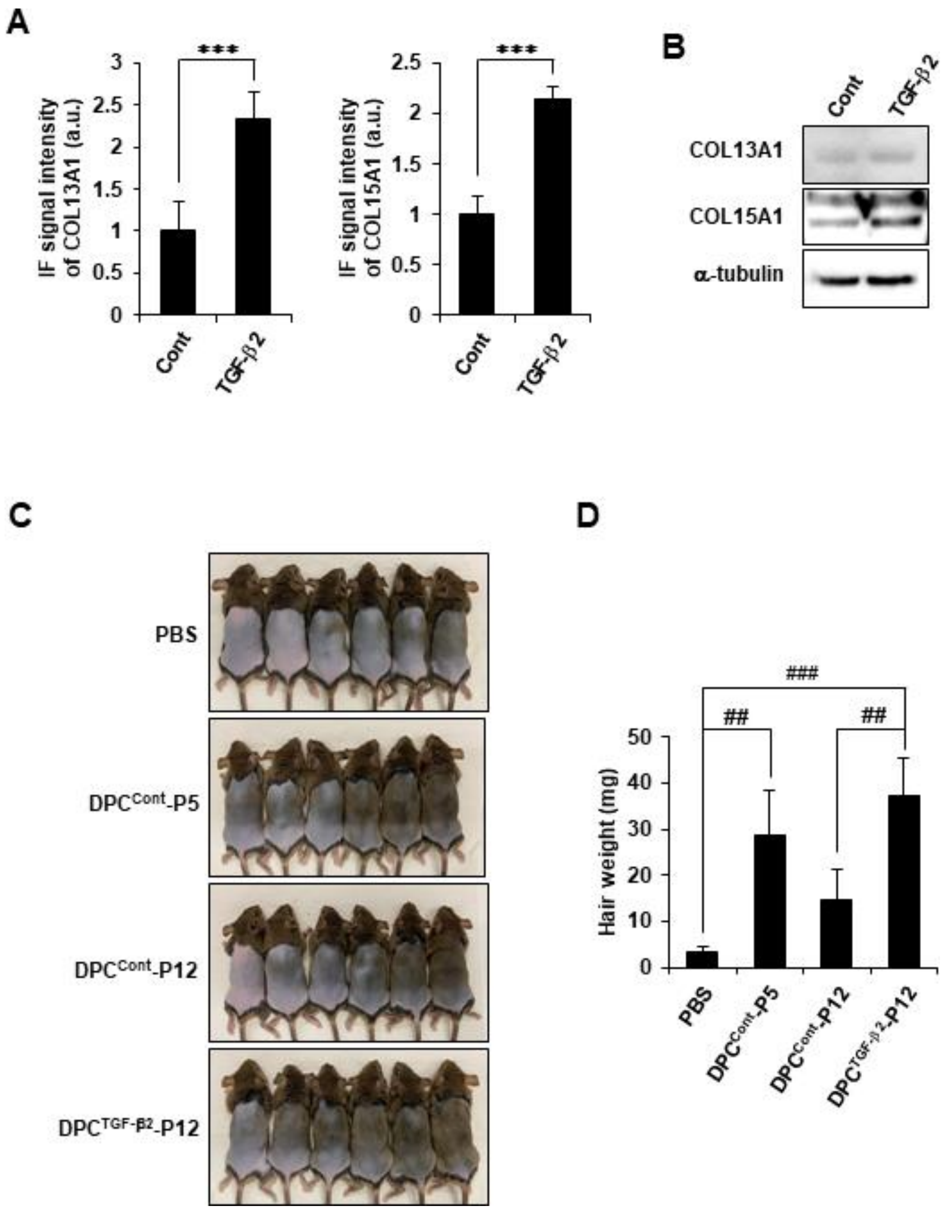

Supplementary Figure 4. TGF-β2-treated hDPCs increase collagen expression and promote hair growth. (**A**) Quantification of COL13A1 and COL15A1 signal intensity in 3D spheroid. (**B**) The expression of COL13A1 and COL15A1 was determined by Western blotting. Equal protein loading was verified by α-tubulin. (**C**, **D**) TGF-β2 -treated hDPCs or untreated hDPCs were injected into the dorsal skin of shaved mice. Photograph was taken (**C**), and hair weight measured (**D**) 14 days later. All error bars indicated the standard error of the mean (SEM). ***$p < 0.005$; $^{\#\#}p < 0.01$; $^{\#\#\#}p < 0.001$. Asterisk indicates Student's *t*-test. Sharp indicates one-way ANOVA

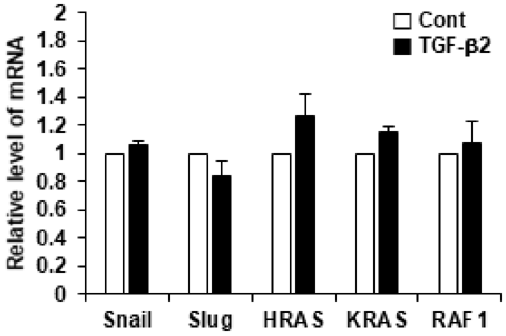

Supplementary Figure 5. TGF-β2 dose not stimulate oncogene expression. Snail, Slug, HRAS, KRAS, and Raf-1 mRNA expression by qRT-PCR in hDPCs (P11) cultured in the absence or presence of TGF-β2.
