# Supplementary material for: TGF-β2 and collagen play pivotal roles in the spheroid formation and anti-aging of human dermal papilla cells
Source: Aging (Albany NY). 2021 Aug 17;13(16):19978–95. doi: 10.18632/aging.203419 (PMC8436940; doi:10.18632/aging.203419)
Supplement: Supplementary Table 1 [file aging-13-203419-s002.pdf]

SUPPLEMENTARY TABLE

Supplementary Table 1. Enriched GO terms and KEGG pathway that are associated with ECM in top list.

Analysis	Term	Count	-LogP**	Fold enrichment
**Gene Ontology Analysis(David Bioinformatics Resources 6.8, NIAID/NIH)**				
**GO_BP**	GO:0030198 extracellular matrix organization	28	8.40	3.85
	GO:0030574 collagen catabolic process	14	6.30	5.90
	GO:0022617 extracellular matrix disassembly	14	5.41	4.97
	GO:0007160 cell-matrix adhesion	14	4.58	4.19
	GO:0007155 cell adhesion	33	3.32	1.94
**GO_MF**	GO:0005518 collagen binding	9	2.93	4.23
	GO:0005201 extracellular matrix structural constituent	8	2.03	3.37
**GO_CC**	GO:0031012 extracellular matrix	30	6.44	2.97
	GO:0005578 proteinaceous extracellular matrix	26	5.25	2.84
	GO:0005615 extracellular space	74	4.29	1.61
	GO:0005581 collagen trimer	13	4.18	4.14
	GO:0009986 cell surface	33	2.72	1.78
**KEGG pathway**				
	Hsa04512 ECM-receptor interaction	16	5.71	4.47
	Hsa04510 Focal adhesion	23	4.49	2.71

GEO data (GSE44765; GSM10990247-9 for test group, GSM109024-3 for control group) was processed with Limma package in R. GO and KEGG pathway were analyzed by using David Bioinformatics resources 6.8. -LogP = Log10P. Value. **$p < 0.01$.
